# Screening for the impact of social media use on self-harm crisis presentations in children and adolescents - are we adhering to NICE guidelines?

**DOI:** 10.1192/j.eurpsy.2026.10563

**Published:** 2026-07-31

**Authors:** A. Daud, A. Tavares, T. Mehdi

**Affiliations:** CAMHS, Berkshire Healthcare NHS Foundation Trust, Reading, United Kingdom

## Abstract

**Introduction:**

National Institute of Clinical Excellence (NICE) guidelines recommend that children and adolescents presenting with self-harm have a psychosocial assessment that includes an enquiry of social media, focussing on its use to connect with others, and any effects on mental health (NICE, 2022). This is supported by evidence of associations between social media use and self-harm (Chen *et al.* Psychiatry Research 2024; 338 115991) including temporal associations between exposure to specific themes and self-harm presentations (Hamilton *et al.* JAACAP Open 2025; 3 431-438). We present a clinical audit of cases referred to our crisis team in England.

**Objectives:**

To complete a clinical audit of assessments of self-harm cases, assessing adherence to NICE guidelines as part of clinical governance to inform the state of assessment quality within our service.

**Methods:**

Retrospective review of cases referred over a month. We included children and adolescents 5-17 presenting with self-harm who underwent a full assessment. We adopted the NICE definition of self-harm - “self-poisoning, self-injury, suicide attempts and non-suicidal self-injury” (NICE, 2025). We used the NHS England definition of social media - any online medium allowing communication and interaction between people, groups or organisations for a variety of reasons, including dissemination of information (NHS England, 2025). We excluded cases where a full assessment was not conducted. We collected data on 1. type of self-harm 2. if social media was enquired about, 3. if social media was used to connect with others and 4. if social media was thought to impact on mental health.

**Results:**

Out of 103 referrals, 50 were for self-harm. Table 1 shows the types of self-harm, with the most common being self-poisoning (48%). In terms of adherence to guidelines: A. Social media enquiry - 72% compliance, B. Social media used to connect with others - 14% and C. Social media impact on mental health - 8%, see figure 1 and table 2. Of the 72% of compliant cases, 97% used social media. While only 8% of assessments explored the impact of social media on mental health, 100% of these assessments revealed a negative impact from social media use.

**Image 1:**
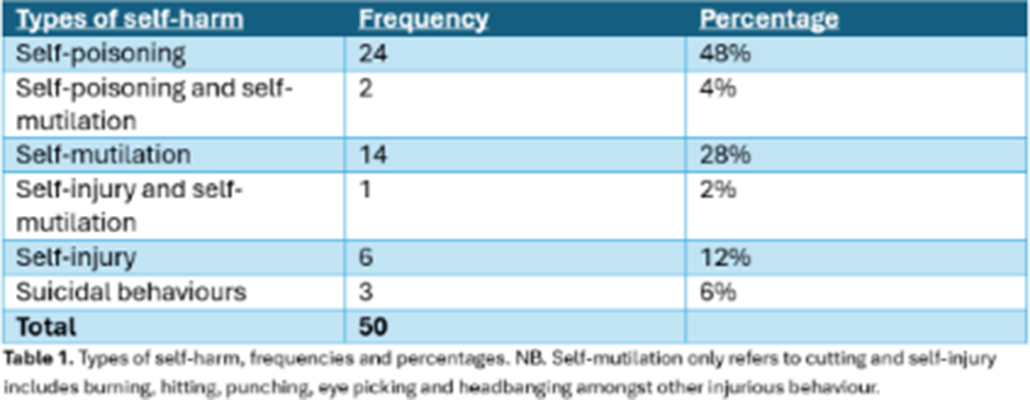
Image 1: Long description.

**Image 2:**
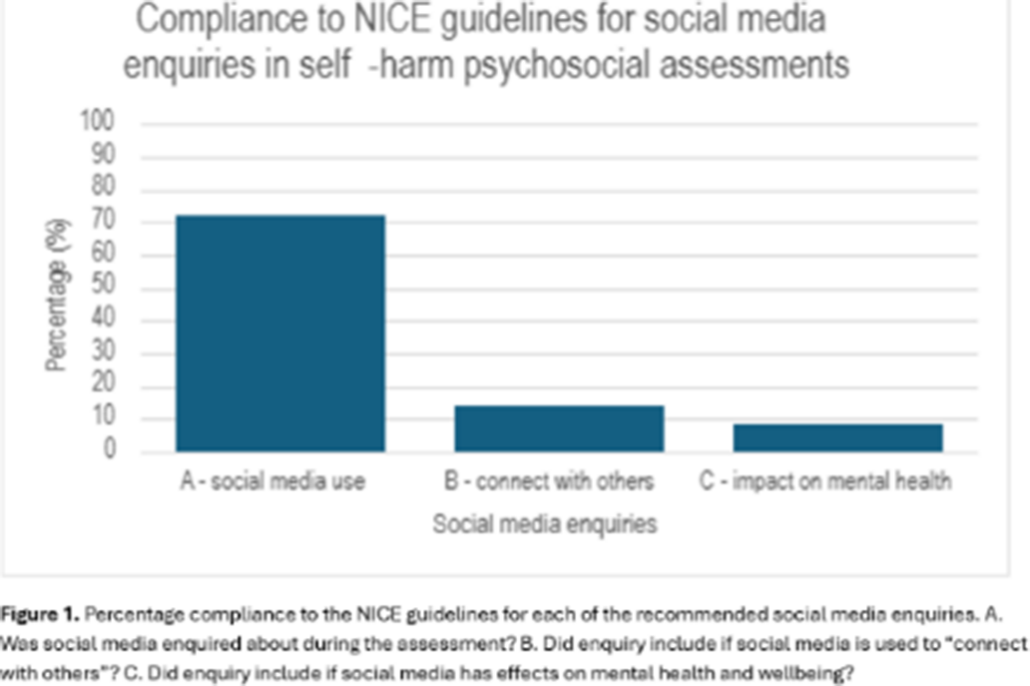

**Image 3:**
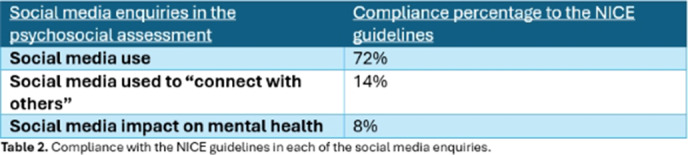

**Conclusions:**

Our audit demonstrated deviation from NICE standards in our service. Lower compliance with secondary questions (14% and 8%) could reflect an underappreciation of the relevance of in-depth exploration of social media use. To complete this audit cycle, we will present results, provide training and complete a reaudit. 35 (97% of those asked) children and adolescents who self-harmed were accessing social media, with 4 (100% of those asked) reports of social media impacting on mental health. Therefore, two strands of further work are indicated - research into the association between social media use and self-harm and quality improvement work to improve adherence to guidelines.

**Disclosure of Interest:**

None Declared

## Long descriptions

### Image 1: Long description

The table consists of three columns: Types of self-harm, Frequency, and Percentage.

* Self-poisoning: 24 frequency, 48 percent.

* Self-poisoning and self-mutilation: 2 frequency, 4 percent.

* Self-mutilation: 14 frequency, 28 percent.

* Self-injury and self-mutilation: 1 frequency, 2 percent.

* Self-injury: 6 frequency, 12 percent.

* Suicidal behaviours: 3 frequency, 6 percent.

* Total: 50 frequency.

A note below the table specifies that self-mutilation refers only to cutting, while self-injury includes burning, hitting, punching, eye picking, and headbanging among other injurious behaviors.

Navigate back to Image 1:.

